# The plasma lipidome of the Quaker parrot (*Myiopsitta monachus*)

**DOI:** 10.1371/journal.pone.0240449

**Published:** 2020-12-01

**Authors:** Hugues Beaufrère, Sara M. Gardhouse, R. Darren Wood, Ken D. Stark

**Affiliations:** 1 Department of Clinical Studies, Ontario Veterinary College, University of Guelph, Guelph, Ontario, Canada; 2 Health Sciences Centre, Ontario Veterinary College, University of Guelph, Guelph, Ontario, Canada; 3 Department of Pathobiology, Ontario Veterinary College, University of Guelph, Guelph, Ontario, Canada; 4 Department of Kinesiology, University of Waterloo, Waterloo, Ontario, Canada; University of Alberta, CANADA

## Abstract

Dyslipidemias and lipid-accumulation disorders are common in captive parrots, in particular in Quaker parrots. Currently available diagnostic tests only measure a fraction of blood lipids and have overall problematic cross-species applicability. Comprehensively analyzing lipids in the plasma of parrots is the first step to better understand their lipid metabolism in health and disease, as well as to explore new lipid biomarkers. The plasma lipidome of 12 Quaker parrots was investigated using UHPLC-MS/MS with both targeted and untargeted methods. Targeted methods on 6 replicates measured 432 lipids comprised of sterol, cholesterol ester, bile acid, fatty acid, acylcarnitine, glycerolipid, glycerophospholipid, and sphingolipid panels. For untargeted lipidomics, precursor ion mass-to-charge ratios were matched to corresponding lipids using the LIPIDMAPS structure database and LipidBlast at the sum composition or acyl species level of information. Sterol lipids and glycerophospholipids constituted the majority of plasma lipids on a molar basis. The most common lipids detected with the targeted methods included free cholesterol, CE(18:2), CE(20:4) for sterol lipids; PC(36:2), PC(34:2), PC(34:1) for glycerophospholipids; TG(52:3), TG(54:4), TG(54:5), TG(52:2) for glycerolipids; SM(d18:1/16:0) for sphingolipids; and palmitic acid for fatty acyls. Over a thousand different lipid species were detected by untargeted lipidomics. Sex differences in the plasma lipidome were observed using heatmaps, principal component analysis, and discriminant analysis. This report presents the first comprehensive database of plasma lipid species in psittacine birds and paves the way for further research into blood lipid diagnostics and the impact of diet, diseases, and drugs on the parrot plasma lipidome.

## Introduction

Dyslipidemias and lipid-accumulation disorders, such as atherosclerosis, hepatic lipidosis, fatty tumors and obesity, are extremely common in captive Psittaciformes [1–4]. The prevalence of severe atherosclerotic lesions in the general parrot population is approximatively 7%, but can be as high as 50% in older parrots [3]. Hepatic lipidosis is also prevalent and one of the major liver diseases in parrots with an estimated overall prevalence of 6%, but susceptible species such as Quaker parrots have an estimated prevalence of 20% [2]. The prevalence of female parrot reproductive disorders associated with upregulated vitellogenesis (hepatic lipid synthesis and lipid transport to eggs) is unknown, but is suspected to be enormous based on clinical experience. Taken together, lipid-related disorders are likely one of the most common causes of non-infectious diseases in captive parrots. However, despite the frequency of these diseases, there is a vast gap of knowledge in regards to the pathophysiology of most of these diseases as well as diagnostic tests, biomarkers, treatment, and therapeutic targets. As dyslipidemic changes are frequently concurrent and comorbid to lipid-accumulation disorders as well as important risk factors, blood lipid analysis can be used for the diagnosis, screening, and monitoring of a variety of diseases associated with lipid dysmetabolism [1, 5–7].

Lipids are the main biomolecular constituents of plasma [8] and are transported in the form of macromolecular aggregates of mixed lipid species and proteins (lipoproteins). Traditionally, dyslipidemias in mammals have been understood as primarily associated with elevated total cholesterol, triglycerides and changes in cholesterol lipoprotein fractions [9, 10]. While psittacine lipoproteins have been measured in plasma using reference methods [11–13], routine laboratory methods have not been validated in parrots and are likely to perform poorly due to the marked differences between avian and mammalian lipoprotein structure and metabolism [14, 15]. For these reasons, lipoprotein testing has not been widely applied to parrots with dyslipidemia or lipid-related disorders and the lipoprotein profiles of psittacine spontaneously-occurring dyslipidemia have not been characterized.

In order to elucidate the pathophysiology of the many common lipid-related diseases in parrots as well as to provide new tools for biomarkers discovery, a more comprehensive analysis of their lipid metabolism in health and disease is required. Comprehensive lipid analysis by mass spectrometry is known as lipidomics. This approach is revolutionizing the way lipid metabolic disorders are investigated as a results of the vast amount of data generated when blood lipids are analyzed using lipidomics [16–20]. Further, in the context of dyslipidemia, specific lipid species of complex lipids may be better targets or biomarkers than crude measurements of a single lipid molecule such as cholesterol (the cholesterol test measures both free cholesterol and the cholesterol moiety of a variety of cholesteryl esters) and triglycerides (the triglyceride test only measures the glycerol backbone of the molecule). Lipidomics allows for the measurement of each lipid in their native biological form. Therefore, thousands of species of fatty acyls, triglycerides, phospholipids, sphingolipids, and cholesteryl esters with a variety of saturated and unsaturated fatty acid chains can be measured in plasma. In humans, lipidomics has been used to study the lipidome [8, 21], dyslipidemia [22, 23], various clinical lipid-accumulation disorders [17, 19, 20], and statin pharmacology [24, 25]. However, this powerful analytical technique has not been applied to psittacine birds or used in avian health research as far as the authors know. Plasma lipidomic profiling in psittacine birds may not only provide a clearer picture of ongoing lipid abnormalities, but also lead to innovation in lipid biomarkers and therapeutic targets beyond cholesterol for a variety of lipid-related diseases.

The first step in applying clinical lipidomics in psittacine medicine is to report the plasma lipidome as blood is the most accessible tissue and plasma biomarkers are the most practical to develop. Knowing the normal lipidome may also allow to detect specific lipidomic signatures of lipid-related diseases in birds. The Quaker parrot (*Myiopsitta monachus*) has been used as an experimental model of lipid disorders in Psittaciformes for dyslipidemia and atherosclerosis [11, 12, 26, 27]. Quaker parrots are also extremely prone to spontaneously-occurring dyslipidemia and lipid-accumulation disorders, more than other psittacine species [2, 4]. It is therefore logical to first use this species to report the psittacine plasma lipidome. The objective of this observational study was to comprehensively report the plasma lipidome of young male and female healthy Quaker parrots using a variety of quantitative (targeted lipidomics) and semi-quantitative (untargeted lipidomics) methods.

## Materials and methods

### Animals and sample collection

Twelve approximately 1-year-old Quaker parrots (*Myiopsitta monachus*) were used for this study. The parrots were captive-bred and hand raised at the Hagen Avicultural Research Institute (QC, Canada). The parrots included 6 males and 6 females; sex was confirmed by DNA testing on blood. The parrots were housed together at the University of Guelph–Central Animal Facility in a large stainless-steel aviary with food and water provided *ad libitum*, and fed a pelletized diet (Tropican 2mm pellet, Hagen Inc., Baie d’Urfee, QC, Canada). They were considered healthy and free of dyslipidemia based on a recent physical examination, CBC, plasma biochemistry, lipoprotein panel, and avian chlamydiosis PCR testing. Animal utilization protocols (AUP) were approved for this research by the University of Guelph—Animal Care Committee (AUP#3875 and AUP#4035).

The parrots were fasted overnight prior to sample collection. Blood was collected and stored according to guidelines for plasma lipidomics [28]. To minimize stress and exertion, birds were captured in the dark and blood was collected within 2–5 minutes following capture. A 1 mL blood sample was collected from each parrot from the right jugular vein under manual restraint using a 3mL syringe connected to a 26g needle. Blood was transferred to a heparinized tube without a serum separator (BD Microtainer, Becton and Dickinson, Mississauga, ON, Canada). Tubes were inverted a minimum of 5 times and placed on ice. Blood was centrifuged for 10 minutes at 1500g and approximately 0.5 mL plasma harvested and aliquoted in cryovials. The plasma was stored at -80C until shipping on dry ice to the various analytical laboratories.

All samples were analyzed by The Metabolomics Innovation Centre (co-located at the University of Alberta and University of Victoria, Canada). Six samples (3 females, 3 males) were submitted to the University of Victoria Genome BC Proteomics Centre for untargeted lipidomics and targeted panels for bile acids, sterols, non-esterified fatty acids, acyl carnitines, and sphingolipids. Another six samples (3 females, 3 males) were submitted to the University of Alberta for the targeted panels using a metabolomics kit for glycerolipids, glycerophosphocholines and cholesteryl esters at the University of Alberta.

### Nutritional analysis

A 100g sample of the pelleted parrot diet was submitted to an independent laboratory (SGS Canada Inc. Agriculture and Food, Mississauga, ON, Canada) for nutritional analysis. Total fat was analyzed by an acid hydrolysis method (SGS-Canada, test QAM-105) and fatty acid composition was obtained using gas chromatographic methods [Association of Official Analytical Collaboration (AOAC) International 991.39, AOAC 963.2].

### Targeted lipidomics

All lipid species were analyzed at the brutto (sum composition) or medio (fatty acyl chain) level of identification [29].

For analysis of bile acids, sterols, steroids, fatty acids, carnitines, and sphingolipids, an Agilent 1290 UHPLC system coupled to an Agilent 6495 QQQ (Agilent, Santa Clara, CA, USA) or a Sciex 4000 QTRAP (Sciex, Framingham, MA, USA) mass spectrometer equipped with an electrospray ion (ESI) source was used. The MS instruments were operated in multiple-reaction monitoring (MRM) with negative-ion (-) detection for analysis of fatty acids and bile acids, and with positive-ion (+) detection for analysis of carnitines, sphingolipids, sterols and steroids.

#### Analysis of bile acids

Bile acids was quantitated by UPLC-MRM/MS on an Agilent 1290 UHPLC system coupled to an Agilent 6495 QQQ mass spectrometer (Agilent, Santa Clara, CA, USA), according to a previously published procedure by the University of Victoria Genome BC Proteomics Centre [30, 31]. A mixed standard solution containing reference substances of 62 bile acids, which were detailed in the same studies [30, 31], was prepared in 50% methanol at 10 nmol/mL for each compound and was used as standard solution S1. This solution was further diluted step by step at a dilution ratio of 1 to 4 (v/v) to have standard solutions of S2 to S10. Fifty μL of S1 to S10 was mixed with 50 μL of a solution containing 14 D-labeled bile acids as internal standard. Twenty μL of each solution was injected to run UPLC-(-)ESI-MRM/MS. Linear-regression calibration curves were constructed using analyte-to-internal standard peak area ratios (As/Ai) versus molar concentrations (nmol/mL) of each bile acid. For the bile acids without isotope-labeled analogues as internal standard, glycodeoxycholic acid-D4 was used as a common internal standard.

For sample preparation, 50 μL of plasma was mixed with 50 μL of the internal standard solution and 400 μL of methanol in an Eppendorf tube. After vortex mixing for 15 s and sonication for 2 min in an ice-water bath, the tube was centrifuged at 15,000 rpm for 15 min in an Eppendorf 5420R centrifuge to pellet proteins. The supernatant was taken out and dried in a nitrogen evaporator under a gentle nitrogen gas flow. The residue was dissolved in 100 μL of 50% methanol. After centrifugal clarification, 20 μL was injected for detection and quantitation of bile acids by UPLC-MRM/MS.

Concentrations of bile acids in each sample were calculated from the standard curves of the individual analytes.

#### Analysis of sterols including cholesterol

A mixed standard solution containing reference substances of 10 sterols (lanosterol, zymosterol, 7-dehydrodesmosterol, desmosterol, dihydrolanosterol, zymostenol, lathosterol, 7-dehydrocholesterol, dihydrolathosterol and cholesterol) which were obtained from Sigma-Aldrich (Oakville, ON, Canada) or Steraloids Inc. (Newport, RI, USA) was prepared in acetonitrile at 10 nmol/mL for each compound, and was used as standard solution S1. This solution was further diluted step by step at a dilution ratio of 1 to 4 (v/v) to have standard solutions of S1 to S10.

Ten μL of plasma was mixed with 90 μL of methanol. After vortex mixing for 15 s and sonication for 2 min in an ice-water bath, the tube was centrifuged at 15,000 rpm for 15 min in an Eppendorf 5420R centrifuge. The supernatant was taken out and dried in a nitrogen evaporator under a gentle nitrogen gas flow. 50 μL of acetonitrile was added to resuspend the residue.

Each sample solution or 50 μL of each standard solution, was mixed with 20 μL of an internal standard solution containing ^13^C_3_-cholesterol. The mixture was subjected to chemical derivatization with 20 mM of dansyl chloride in the presence of 100 mM 4-(dimethylamino)-pyridine (DMAP) as a catalyst at 50 °C for 60 min, according to a previously published procedure with necessary modifications [32]. After derivatization, 20 μL of each resultant solution was injected to run UPLC-(+)ESI-MRM/MS on a C18 UPLC column (2.1 x 50 mm,1.7 μm) with 0.1% formic acid and isopropanol/acetonitrile (1:2) as the mobile phase for binary-solvent gradient elution, at a flow of 0.4 mL/min and 60 °C.

Linear-regression calibration curves were constructed using analyte-to-internal standard peak area ratios (As/Ai) versus molar concentrations (nmol/mL) of the standard solutions for each sterol with 13C3-cholesterol as a common internal standard. Concentrations of sterols detected in each sample were calculated from the standard curves of the individual analytes.

#### Analysis of selected steroids

A mixed standard solution containing reference substances of cortisol, aldosterone and androstenedione (Steraloids Inc., Newport, RI) was prepared in methanol at 10 nmol/mL for each compound. This solution was further diluted step by step at a dilution ratio of 1 to 4 (v/v) with the same solvent to have standard solutions of S1 to S9.

Fifty μL of plasma was mixed with 425 μL of 125 mM sodium acetate buffer (pH = 5.5) and 25 μL of an internal standard solution containing cortisol-D4 and 6β-OH cortisol-D4. After vortex-mixing, 1 mL of ethyl acetate was added and the mixture was vortex-mixed for 30 s followed by centrifugal clarification. The clear supernatant was taken out and dried in a nitrogen evaporator. The residue was reconstituted in 50 μL of methanol. Ten μL was injected to run UPLC-(+)ESI-MRM/MS on a C18 UPLC column (2.1 x 100 mm, 1.7 μm) with 0.1% formic acid and acetonitrile as the mobile phase for binary-solvent gradient elution, at 0.3 mL/min and 50 °C.

Linear-regression calibration curves were constructed using analyte-to-internal standard peak area ratios (As/Ai) versus molar concentrations (nmol/mL) of the standard solutions. Concentrations of steroids detected in each sample were calculated from the standard curves of the individual analytes, with internal calibration.

#### Analysis of fatty acids

Quantitation of fatty acids was performed using 3-nitrophenylhydrazine (3-NPH) derivatization–UPLC-MRM/MS, adapted from a published procedure from Han et al. [33]

A mixed standard solution containing reference substances of 46 C_2_ to C_24_ saturated and unsaturated fatty acids (as listed in Tables 5–7) and 9 organic acids of the tri-carboxylic acid cycle (glycolic, lactic, malic, succinic, fumaric, citric, isocitric, pyruvic and α-ketoglutaric acid), which were obtained from Sigma-Aldrich or from Cayman Chemical (Arbor, Michigan, USA), all the targeted organic acids was prepared in methanol at 200 nmol/mL for each compound. This solution was further diluted step by step at a dilution ratio of 1 to 5 (v/v) to have standard solutions of S1 to S10.

Ten μL of each plasma was mixed with 90 μL of methanol. After vortex-mixing for 15 s, 3-min sonication and centrifugal clarification for 15 min, 50 μL of the supernatant, or 50 μL of each standard solution was mixed with 20 μL of a solution containing deuterium-labeled analogues of C8 to C24 even-carbon saturated fatty acids as internal standard, 25 μL of 200-mM 3-NPH solution and 2 μL of 150-mM EDC solution. The mixture was allowed to react at 35 °C for 60 min in a thermomixer at a shaking frequency of 900 rpm. After the reaction, 10 μL was injected onto a C8 UPLC column (2.1 mm I.D. x 100 mm, 1.7 μm) for LC separation with a mobile phase composed of 1 mM ammonium fluoride in water and isopropanol-acetonitrile for binary-solvent gradient elution at 0.4 mL/min and 55 °C. The efficient gradient was 10% to 100% in 14 min.

Linear-regression calibration curves were constructed using analyte-to-internal standard peak area ratios (As/Ai) versus molar concentrations (nmol/mL) of the standard solutions for each compound. Concentrations of organic acids detected in each sample were calculated from the standard curves of the individual analytes with internal calibration.

#### Analysis of carnitines

Quantitation of carnitines was carried out according to a previously published method by Han et al [34].

A mixed standard solution containing reference substances of 28 free and acyl carnitines, as previously described [34], was prepared in methanol at 10 nmol/mL for each compound. This solution was further diluted step by step at a dilution ratio of 1 to 4 (v/v) to have standard solutions of S1 to S9.

Fifteen μL of each plasma was mixed with 85 μL of methanol. After vortex-mixing for 30 s and sonication for 3 min, followed by centrifugal clarification at 15,000 rpm for 15 min, 50 μL of the supernatant was mixed with 50 μL of 100 mM of 3-NPH solution and 50 μL of a mixed solution containing 100 mM of EDC, HCl and 3% pyridine, all in 75% aqueous methanol. The mixture was allowed to react at 30 °C for 30 min in a thermomixer at a shaking frequency of 900 rpm. After the reaction, 50 μL of ^13^C_6_-3NPH derivatives of all the targeted carnitines, which was in advance prepared in a “one-pot” reaction was added. After mixing, 20 μL of each solution was injected for quantitation of free and acyl carnitines in the samples by UPLC-MRM/MS with positive-ion detection using the previously described method [34]. A C8 UPLC column (2.1 x 100 mm, 1.8 μm) was used for LC separation with water-0.1% formic acid and acetonitrile as the mobile phase for binary gradient elution.

Linear-regression calibration curves were constructed using analyte-to-internal standard peak area ratios (As/Ai) versus molar concentrations (nmol/mL) of the standard solutions for each bile acid. Concentrations of carnitines in each sample were calculated from the calibration curves of the individual carnitines. Concentrations of organic acids detected in each sample were calculated from the standard curves of the individual analytes with internal calibration.

#### Analysis of sphingolipids

A mixed standard solution containing reference substances of 60 sphingolipids (from Avantis Polar Lipids, Alabaster, AL, USA; or Cayman chemical) was dissolved in methanol at 50 nmol/mL for each compound. This solution was further diluted step by step at a dilution ratio of 1 to 4 (v/v) to have standard solutions of S1 to S10.

20 μL of plasma was mixed with 180 μL of methanol-chloroform (1:1). After vortex-mixing for 30 s and sonication for 15 min at 15000 rpm. The supernatant was taken out and dried down in a nitrogen evaporator. The residue was resuspended in 50 μL of methanol.

10 μL of each standard solution or each sample solution was injected onto a C8 UPLC column (2.1 x 50 mm, 1.7 μm) for UPLC-MRM/MS with positive-ion detection, with 0.1% formic acid in water (A) and acetonitrile-isopropanol (1:1) (B) as the mobile phase for binary gradient elution, at 0.4 mL/min and 55 °C. The efficient gradient is 50% to 100% B in 15 min.

Linear-regression calibration curves were constructed using peak areas versus molar concentrations (nmol/mL) of the standard solutions for each sphingolipid. Concentrations of each detected lipid were calculated from the calibration curves with peak areas. For those sphingolipids detected but without the standard substances available, their concentrations were estimated using the calibration curves from one of the homologues in each sphingolipid class with the closest carbon number of their acyl chains.

#### Analysis of glycerolipids, glycerophospholipids, and cholesteryl esters

For these analytes, a metabolomics kit (Absolute IDQ p400 HR kit, Biocrates Life Sciences AG, Innsbruck, Austria) was used according to the manufacturer standard operating procedure using UHPLC with a C18 column coupled to a QExactive HF OrbiTrap mass spectrometer (Thermo Fisher Scientific, Waltham, MA, USA) with protocols previously published by the University of Alberta—The Metabolomics Innovation Centre [35, 36].

Only lipid metabolites were reported. The kit panel overlapped with the other targeted panels for carnitines and some sphingolipids. Only the analytes not measured by other targeted techniques were reported.

### Untargeted lipidomics

A Dionex Ultimate 3000 UHPLC system coupled to a Thermo Scientific LTQ-Orbitrap Velos Pro mass spectrometer equipped with an electrospray ionization (ESI) source was used.

Fifty μL of plasma was aliquoted to a 1.5-mL Eppendorf safe-lock tube. 250 μL of mixed methanol/chloroform (3:1) was added. The tube was vortex mixed for 20 s at 3000 rpm, sonicated in an ice-water bath for 3 min and then centrifuged at 15000 rpm for 20 min. The clear supernatant was taken out and transferred to an LC injection micro-vial. 10-μL aliquot were injected to run reversed-phase LC-MS for detection and relative quantitation of lipids, in (+) and (-) ion modes, respectively, with two rounds of LC-MS runs.

UHPLC-MS/MS runs were carried out for analysis of various lipids with the use of a C8 UHPLC column (2.1 x 50 mm, 1.7 μm) for chromatographic separation. The mobile phase was (A) 0.02% formic acid in water and (B) 0.02% formic acid in acetonitrile-isopropanol (1:1, v/v). The efficient gradient was 5% to 50% B in 5 min; 50% to 100% B in 15 min and 100% B for 2.5 min before the column was equilibrated for 4 min at 5% B between injections. The column flow was 400 μL/min and the column temperature was maintained at 55 °C. The MS instrument was operated in the survey-scan mode with full-mass and high-resolution Fourier transform MS detection at a mass resolution of 60,000 FWHM @ m/z 200. The mass scan range was m/z 70 to 1800 for both positive-ion and negative-ion detection. Along with the MS data, MS/MS data was also acquired using collision induced dissociation (CID) with top 6 acquisitions.

Two MS full-mass detection datasets were acquired. To process these MS datasets, the raw data files were converted to a common data format and were processed with XCMS (https://xcmsonline.scripps.edu/) in R for peak detection, retention time shift corrections, peak grouping and peak alignment. Mass de-isotoping and removal of chemical background noise peaks were performed, with partial manual interventions based on several rules in chemistry and mass spectrometry.

Lipid annotations were performed manually using the LIPIDMAPS structural database (LMSD) bulk structures [37] and LipidBlast software (Lipidblast-mz-lookup-v49 module) [38] for positive mode [(M+H)+ and (M+Na)+ ions] and for negative mode [(M-H)- ions]. A m/z error of 5ppm (Lipidmaps) or 0.008 Da (Lipidblast) was used. Only biologically relevant species were included in the search. LipidBlast was used to query glycerolipids (DG, TG), glycerophospholipids (PC, PE, PS, PG, PI, PA) and sphingolipids (SM, gangliosides, ceramides). LMSD was used to additionally query MG, cholesteryl esters (CE), and fatty acyls (search restricted to fatty acids and acyl-carnitines). Duplicate hits were removed manually. The LIPIDMAPS nomenclature was used for lipid names, category names, and abbreviations. Lipid species were reported at the brutto level of information [29].

### Statistical analysis

Targeted lipidomics data were reported with their median, interquartile range (IQR), minimum, and maximum for each lipid group and relative percentage for some lipid groups. A global barplot on polar coordinate was performed with all targeted panels to have a graphical representation of the whole lipidome. For untargeted lipidomics data, only the main lipid species per lipid groups were reported with the most common lipid species based on peak intensity.

For differences between sexes, targeted lipidomics data were pre-processed using auto scaling (mean centered and divided by standard deviation) for multivariate analysis. Metabolites below the limits of detection were removed from the analysis. Differences in individual lipid metabolites between sexes were then assessed using serial t-tests with a false discovery rate of 5%. The lipidome was explored using principal component analysis (PCA) to detect sex lipidomics signatures using an unsupervised technique. Lipid analytes contribution to respective principal components was assessed by evaluation of the PCA biplots. Differences between sexes were further explored using a supervised classifying multivariate tool: partial least squares—linear discriminant analysis (PLS-DA). Loading plots were also inspected for important features as well as the variable importance in projection (VIP) scores. A heatmap with hierarchical clustering was also generated for data exploration using the 50 most important analytes based on t-test p-values on normalized data. R (version 4.0.0, R foundation for statistical computing, Vienna, Austria) was used for descriptive statistical analysis, ggplot2 for bar plots [39], and MetaboAnalyst 4.0 for multivariate analysis and graphs [40].

## Results

### Nutritional analysis

The parrot pelleted diet contained 11.5% total fat including 1.6% of saturated fatty acids, 4.8% of monounsaturated fatty acids, and 5.1% of polyunsaturated fatty acids. The fatty acid profile included palmitic acid (FA 16:0) as the preponderant saturated fatty acid, oleic acid (FA 18:1) as the preponderant monounsaturated fatty acid, and linoleic acid (FA 18:2) as the preponderant polyunsaturated fatty acid. The combination of linoleic acid and oleic acid represented more than 80% of total dietary fatty acids. The complete fatty acid profile is reported in Table 1.

**Table 1:** Fatty acid composition of the Quaker parrot pelleted diet.

Parameter	% of total fatty acids
FA(6:0) Caproic acid	<0.1
FA(8:0) Caprylic acid	<0.1
FA(10:0) Capric acid	<0.1
FA(12:0) Lauric acid	<0.1
FA(14:0) Myristic acid	<0.1
FA(14:1) Myristoleic acid	<0.1
FA(15:0) Pentadecylic acid	<0.1
FA(15:1) Pentadecanoic acid	<0.1
FA(16:0) Palmitic acid	9.5
FA(16:1) Palmitoleic acid	<0.1
FA(17:0) Heptadecanoic acid	<0.1
FA(17:1) Cis-10 heptadecenoic acid	<0.1
FA(18:0) Stearic acid	2.6
FA(18:1) Elaidic acid	<0.1
FA(18:1) Oleic acid	41.3
FA(18:2) Trans-linolelaidic acid	<0.1
FA(18:2) Linoleic acid	39.6
FA(18:3) Gamma-linolenic acid	<0.1
FA(18:3) Alpha-linolenic acid	4.8
FA(18:4) stearidonic acid	<0.1
FA(20:0) Arachidic acid	0.5
FA(20:1) Eicosenoic acid	0.6
FA(20:2) Eicosadienoic acid	<0.1
FA(20:3) Cis-Eicosatrienoic acid	<0.1
FA(20:4) Arachidonic acid	<0.1
FA(20:5) Eicosapentaeonic acid	<0.1
FA(21:0) Heneicosanoic acid	<0.1
FA(22:0) Behenic acid	0.7
FA(22:1) Erucic acid	<0.1
FA(22:2) Cis-docosadienoic acid	<0.1
FA(22:4) Docosatetraenoic acid	<0.1
FA(22:5) Docosapentaenoic acid	<0.1
FA(22:6) Docosahexaneoic acid	<0.1
FA(24:0) Lignoceric acid	0.3
FA(24:1) Nervonic acid	<0.1

Limits of quantitation is 0.1%.

### Plasma lipidome

The number of lipid species (not accounting for isomeric species, which are numerous for triacylglycerols and diaryl lipids) quantified by targeted lipidomics panels are reported in Table 2 and included 432 lipids. The highest number of lipids analyzed were for glycerophospholipids, but only included glycerophosphocholines.

**Table 2:** Number of lipid species or group of isomeric species quantified by targeted lipidomics panels in Quaker parrots (*Myiopsitta monachus*) plasma.

Category	Main class	Subclass	N
Fatty acyls	Fatty acids (FA) and conjugates (non-esterified)	Hydroxy fatty acids	1
		Saturated fatty acids	27
		Unsaturated fatty acids	18
	Fatty esters	Fatty acyl carnitines	39
Glycerolipids	Diradylglycerols	Acyl-alkylglycerols	3
		Diacylglycerols (DG)	14
	Triradylglycerols	Triacylglycerols (TG)	38
Glycerophospholipids	Glycerophosphocholines	Monoalkylglycerophosphocholines	4
		Monoacylglycerophosphocholines	13
		Alkyl-acylglycerophosphocholines	35
		Diacylglycerophosphocholines	71
Sphingolipids	Sphingoid bases (non-esterified)	Various subclasses	9
	Ceramides	N-acylsphinganines	13
		N-acylsphingosines	22
	Phosphosphingolipids	Ceramide phosphocholines (sphingomyelins)	17
	Neutral glycosphingolipids	Simple GLc series	9
Sterol lipids	Bile acids and derivatives	Various subclasses	75
	Sterols	Cholesterol and derivatives	10
		Sterol esters	14
Total			432

**Fig 1:**
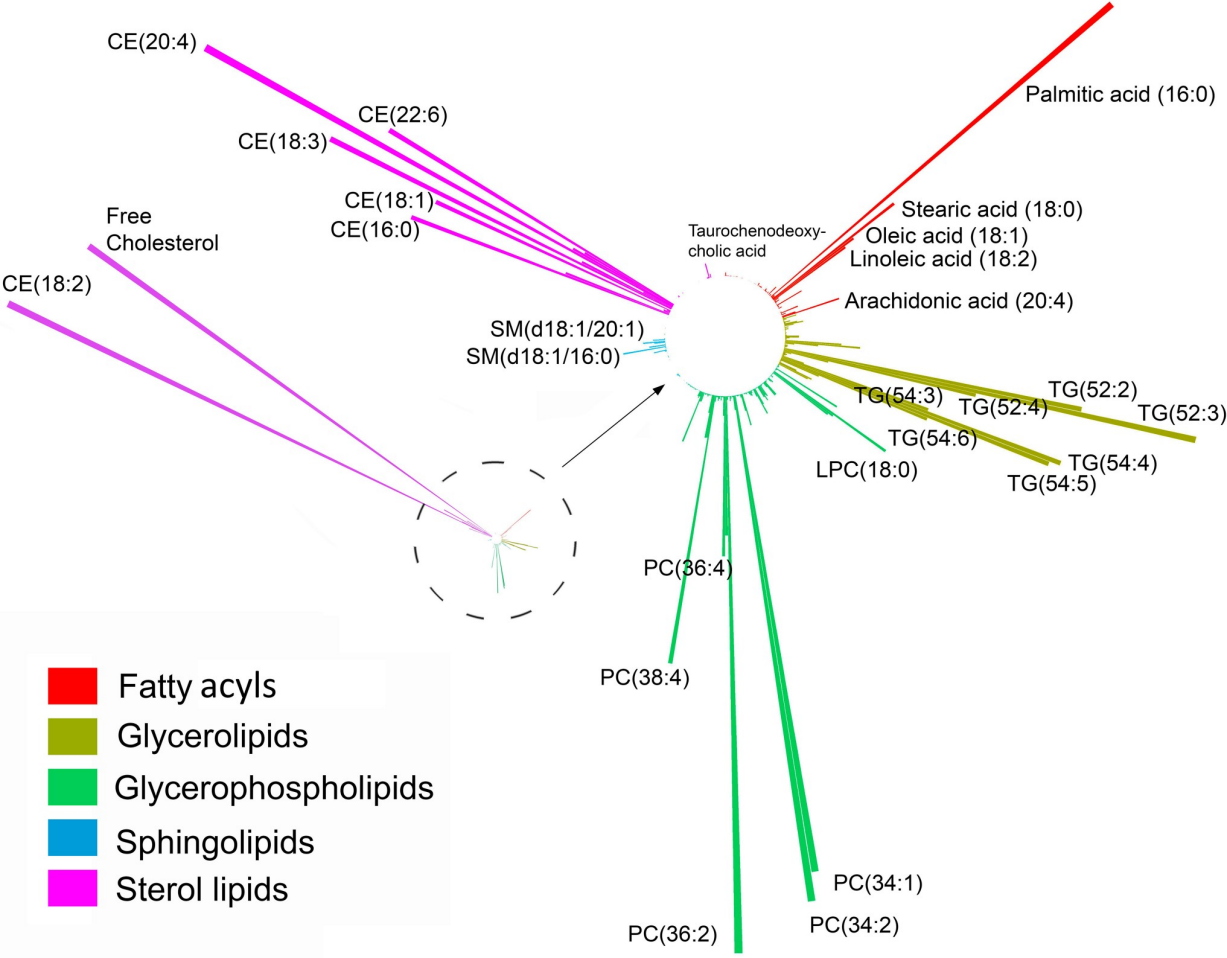
Circular barplot of the mean concentration of lipid species across 5 lipid categories measured by targeted lipidomics in Quaker parrots (*Myiopsitta monachus*). The dashed circle and arrow indicate the zoomed-in portion of the left barplot. Each lipid category is color coded differently. Lipid species abbreviation follows the LIPIDMAPS nomenclature. While lipid species were quantitively measured, comparisons across lipid category should be made carefully.

A global representation of the targeted lipidomics panel is also presented in Fig 1.

The Quaker parrot plasma lipidome, as assessed using these incomplete panels, was dominated by sterol lipids and glycerophospholipids on a molar basis followed by glycerolipids (Fig 1 and Table 3). Cholesterol and its esters were the most abundant lipid species by far especially for free cholesterol and cholesteryl linoleate [CE(18:2)].

**Table 3:** Percentage of lipids by lipid category on a molar basis in the plasma of Quaker parrots (*Myiopsitta monachus*) as determined by targeted lipidomics panels.

Lipid category	%
Fatty acyls	4.8
Glycerolipids	11.3
Glycerophospholipids	14.5
Sphingolipids	0.6
Sterol lipids	68.7

Untargeted lipidomics results yielded a high number of lipid species (Table 4). They included lipid categories and species not represented or measured in the targeted lipidomics panels. The most diverse lipid category was the glycerophospholipids, which was dominated by glycerophosphoethanolamines (PE) and glycerophosphocholines (PC) species. Of those, only PCs were quantified using targeted methods. Identified abundant species did not necessarily correspond to lipid found to be abundant on targeted panels.

**Table 4:** Number of lipid species or group of isomeric/isobaric species identified by untargeted lipidomics in six Quaker parrots (*Myiopsitta monachus*) plasma. Lipid identification was performed using LIPIDMAPS structural database (LMSD) bulk structure and LipidBlast. The most abundant identified lipids are also displayed by category. Lipid species abbreviation follows the LIPIDMAPS nomenclature. Abundant species were determined based on peak intensity within lipid groups.

Category	N	Abundant species
Fatty acyls		
Fatty acids and conjugates (FA)	161	FA(22:6),FA(18:3),FA(20:4),FA(18:2),FA(18:1)
Fatty acyl carnitines (C)	30	C22:6,C18:1
Glycerolipids		
Monoacylglycerols (MG)	8	MG(18:1),MG(18:2)
Diacylglycerols (DG)	0	
Triacylglycerols (TG)	57	TG(52:2),TG(52:3),TG(50:1),TG(54:6),TG(54:5)
Glycerophospholipids		
Glycerophosphates (PA)	118	PA(36:2),PA(34:1),PA(38:2),PA(36:1)
Glycerophosphocholines (PC)	136	LPC(18:2), PC(34:1),PC(34:2), LPC(20:4)
Glycerophosphoethanolamines (PE)	211	PE(36:2),PE(P-18:0)
Glycerophosphoglycerols (PG)	65	PG(36:2),PG(34:1),PG(36:0)
Glycerophosphoinositols (PI)	61	PI(36:2),PI(38:3),PI(34:2),PI(36:1),PI(34:1)
Glycerophosphoserines (PS)	120	PS(21:0),PS(35:1),PS(44:8),PS(40:6)
Sphingolipids		
Ceramides (Cer)	55	Cer(36:2),Cer(24:1),Cer(26:0)
Sphingomyelins (SM)	7	SM(42:1)
Glycosphingolipids	5	[glycan]-Cer(38:1),[glycan]-Cer(34:1),
Sterol lipids		
Cholesteryl esters (CE)	5	CE(22:6), CE(19:0),CE(18:2)
TOTAL	1039	

Raw data for untargeted lipidomics prior to lipid identification and results of the targeted panels were published in the public domain in a permanent scientific data repository (Beaufrère H, 2020, The plasma lipidome of the Quaker parrot (*Myiopsitta monachus*), https://doi.org/10.5683/SP2/XUW31U, Scholars Portal Dataverse, V1).

#### Fatty acyls

Measured free fatty acids included non-esterified saturated fatty acids (Table 5), unsaturated fatty acids (Table 6) of short, medium, long, and very-long chains as well as one hydroxy-fatty acid (Table 7). Non-esterified fatty acids correspond to only a small proportion of all fatty acids in the plasma as most are esterified to various head groups.

**Table 5:** Plasma free saturated fatty acid concentration (μM) in six Quaker parrots (*Myiopsitta monachus*) determined by mass spectrometry.

Lipids	Median	IQR	Min	Max	%
FA(02:0) Acetic acid	5.234	2.191	1.013	6.978	0.5
FA(03:0) Propionic acid	0.019	NA	<LOD	0.348	0.0
FA(04:0) Butyric acid	1.271	0.787	0.912	2.19	0.1
FA(04:0) Isobutyric acid	1.52	0.248	1.133	1.88	0.1
FA(05:0) 2-Methylbutyric acid	1.965	0.306	1.67	2.48	0.2
FA(05:0) Isovaleric acid	0.864	0.178	0.62	1.458	0.1
FA(05:0) Valeric acid	0.051	0.135	0	0.195	0.0
FA(06:0) 3-Methylvaleric acid	0.04	0.033	0	0.073	0.0
FA(06:0) Caproic acid	0.357	0.227	0.186	0.515	0.0
FA(06:0) Isocaproic acid	<LOD	NA	<LOD	0.116	0.0
FA(08:0) Caprylic acid	4.843	1.517	1.325	5.801	0.4
FA(09:0) Pelargonic acid	1.666	0.512	0.814	2.256	0.2
FA(10:0) Capric acid	1.172	0.399	0.918	1.703	0.1
FA(11:0) Undecylic acid	0.19	0.029	0.096	0.21	0.0
FA(12:0) Lauric acid	2.779	0.599	2.319	3.443	0.3
FA(13:0) Tridecylic acid	0.062	0.15	0.017	0.249	0.0
FA(14:0) Myristic acid	17.871	3.756	13.139	20.906	1.6
FA(15:0) Pentadecylic acid	2.918	0.855	2.279	3.428	0.3
FA(16:0) Palmitic acid	744.897	75.048	472.605	1014.033	68.6
FA(17:0) Margaric acid	5.97	1.271	3.534	7.728	0.5
FA(18:0) Stearic acid	264.991	23.737	162.998	311.587	24.4
FA(19:0) Nonadecylic acid	2.699	0.492	2.483	3.702	0.2
FA(20:0) Arachidic acid	8.135	1.556	6.504	9.069	0.7
FA(21:0) Heneicosylic acid	0.867	0.254	0.505	0.964	0.1
FA(22:0) Behenic acid	6.088	1.776	3.312	7.097	0.6
FA(23:0) Tricosylic acid	0.87	0.345	0.577	1.371	0.1
FA(24:0) Lignoceric acid	8.134	1.426	4.36	9.473	0.7

IQR, interquartile range; NA, not applicable; LOD, limits of detection.

**Table 6:** Plasma free unsaturated fatty acid concentration (μM) in six Quaker parrots (*Myiopsitta monachus*) determined by mass spectrometry.

Lipids	Median	IQR	Min	Max	%
FA(14:1) Myristoleic acid	0.506	0.088	0.396	0.635	0.1
FA(16:1) Palmitoleic acid	6.071	1.638	4.186	9.562	1.4
FA(18:1) Oleic acid	173.823	35.534	92.953	234.033	40.0
FA(18:2) Linoleic acid	157.261	40	76.826	210.138	36.2
FA(18:3) α-Linolenic acid	8.921	2.776	6.122	12.686	2.1
FA(20:1) Gondoic acid	1.524	0.397	1.251	1.95	0.4
FA(20:2) Eicosadienoic acid	0.474	0.143	0.324	0.637	0.1
FA(20:3) Dihomo-y-linolenic acid	0.875	0.255	0.633	1.158	0.2
FA(20:4) Arachidonic acid	47.954	11.11	38.127	54.582	11.0
FA(20:5) Eicosapentaenoic acid (EPA)	1.76	0.635	1.381	2.583	0.4
FA(20:6) Eicosahexaenoic acid	1.891	0.628	1.459	2.643	0.4
FA(22:1) Erucic acid	0.17	0.036	0.099	0.184	0.0
FA(22:2) Docosadienoic acid	0.069	0.009	0.047	0.088	0.0
FA(22:3) Docosatrienoic acid	0.078	NA	<LOD	0.324	0.0
FA(22:4) Docosatetraenoic acid	<LOD	NA	<LOD	<LOD	0.0
FA(22:5) Docosapentaenoic acid	1.645	0.369	1.21	1.856	0.4
FA(22:6) Docosahexaenoic acid (DHA)	31.145	6.36	22.717	41.09	7.2
FA(24:1) Nervonic acid	0.16	0.04	0.105	0.189	0.0

IQR, interquartile range; NA, not applicable; LOD, limits of detection.

**Table 7:** Plasma free hydroxy fatty acid concentration (μM) in six Quaker parrots (*Myiopsitta monachus*) determined by mass spectrometry.

Lipids	Median	IQR	Min	Max
FA(16:0-OH) hydroxypalmitic acid	0.1	0.057	0.064	0.2

IQR, interquartile range.

Palmitic acid was the most abundant free fatty acid in the plasma by far followed by stearic acid, oleic acid, linoleic acid, and arachidonic acid (Fig 1). It was also the most abundant free saturated fatty acid representing more than 68% of all plasmatic free saturated fatty acids. Palmitic and stearic acids represented 93% of all saturated fatty acids. Oleic acid was the most common monounsaturated fatty acid, but also the most common unsaturated fatty acid. Linoleic acid was the most abundant polyunsaturated fatty acid and omega-6 fatty acid and docosahexaenoic acid (DHA) the most common omega-3 fatty acid.

Acyl-carnitines were also measured (Table 8). They are activated fatty acids that can be transported across mitochondrial membranes for beta-oxidation to produce energy. Unsurprisingly, acetyl carnitine was the most common acyl carnitines as it facilitates the movement of acetyl-CoA as end-products of mitochondrial fatty acid oxidation. Oleyl-carnitine and linoleyl-carnitine were the most abundant long-chain fatty acyl carnitines.

**Table 8:** Plasma acyl carnitines concentration (μM) in six Quaker parrots (*Myiopsitta monachus*) determined by mass spectrometry.

Lipids	Median	IQR	Min	Max	%
C02:0 Acetyl carnitine	5.77	1.512	3.25	6.78	45.6
C03:0 Malonyl-carnitine	0.411	0.113	0.291	0.552	3.2
C03:0 Propionyl-carnitine	0.347	0.074	0.283	0.433	2.7
C04:0 Butyryl-carnitine	0.578	0.148	0.356	0.681	4.6
C04:0 Hydroxybutyryl-carnitine	0.107	0.055	0.039	0.138	0.8
C04:0 Isobutyryl—Carnitine	0.212	0.072	0.136	0.257	1.7
C04:0 Methylmalonyl-carnitine	0.791	0.266	0.663	0.96	6.2
C04:0 Succinyl-carnitine	0.042	0.024	0.019	0.065	0.3
C05:0 1-3-Methylcrotonyl-L-Carnitine	0.004	0.002	0.003	0.005	0.0
C05:0 2-Methylbutyryl-L-Carnitine	0.326	0.089	0.261	0.44	2.6
C05:0 3-hydroxyisovaleryl-carnitine	0.199	0.048	0.132	0.311	1.6
C05:0 Glutaryl-carnitine	0.328	0.057	0.277	0.404	2.6
C05:0 Isovaleryl-Carnitine	0.346	0.05	0.288	0.4	2.7
C05:0 Valeryl-carnitine	1.4	0.325	0.625	1.62	11.1
C05:1 Tiglyl-carnitine	0.034	0.009	0.026	0.043	0.3
C06:0 Hexanoyl-carnitine	0.056	0.034	0.027	0.084	0.4
C06:0 3-Methylglutaryl-Carnitine	0.003	0.001	0.002	0.004	0.0
C06:1 Hexenoylcarnitine	0.02	0.018	0.01	0.086	0.2
C08:0 Octanoyl-carnitine	0.148	0.107	0.051	0.427	1.2
C08:1 Hydroxyoctenoyl-carnitine	0.444	0.592	0.153	1.11	3.5
C08:1 Octenoyl-carnitine	0.103	0.083	0.009	0.164	0.8
C10:0 Decanoyl-carnitine	0.024	0.007	0.015	0.028	0.2
C10:1 Decenoyl-carnitine	0.024	0.008	0.012	0.03	0.2
C12:0 Dodecanedioyl-carnitine	0.01	0.005	0.001	0.012	0.1
C12:0 Dodecanoyl-carnitine	0.03	0.007	0.025	0.035	0.2
C12:1 Dodecenoyl-carnitine	0.017	0.006	0.014	0.021	0.1
C14:0 Tetradecanoyl-carnitine	0.029	0.01	0.023	0.038	0.2
C14:1 Tetradecenoylcarnitine	0.066	0.02	0.042	0.079	0.5
C14:2 Hydroxytetradecadienoyl-carnitine	0.017	0.004	0.011	0.019	0.1
C14:2 Tetradecadienoyl-carnitine	0.035	0.011	0.021	0.04	0.3
C16:0 3-hydroxyhexadecanoyl-carnitine	0.005	0.002	0.003	0.007	0.0
C16:0 Palmitoyl-carnitine	0.118	0.04	0.091	0.18	0.9
C16:1 Hexadecenoyl-carnitine	0.009	0.005	0.006	0.015	0.1
C16:2 Hexadecadienoyl-carnitine	0.012	0.006	0.009	0.025	0.1
C18:0 Octadecanoyl-carnitine	0.074	0.023	0.053	0.109	0.6
C18:1 Oleyl-carnitine	0.294	0.105	0.192	0.403	2.3
C18:2 Linoleyl-carnitine	0.19	0.054	0.101	0.226	1.5
C20:0 Arachidyl-carnitine	0.03	0.008	0.025	0.036	0.2
C20:4 Arachidonoyl-carnitine	0.011	0.004	0.009	0.015	0.1

IQR, interquartile range.

Fatty acyls also include a large number of lipid mediators, which were also measured in these parrots, but as part of a different study on the plasma mediator lipidome.

#### Glycerolipids

The most abundant glycerolipids were triacylglycerols (Table 9). They were quantified based on identification at the sum composition. The most common species were TGs in C52 or C54 with 2 to 6 double bonds. The four most common TG species represented more than half (57.3%) of all TGs on a molar basis. Untargeted lipidomics also identified these TGs as common. While only the sum composition was used, based on typical stereospecific structures of biological TGs in animals and common plasma free fatty acids from Tables 5 and 6, they likely contained a high proportion of palmitic acid, and C18 fatty acids such as stearic, oleic, linoleic, and alpha-linolenic acids and arachidonic acid as a highly unsaturated fatty acid.

**Table 9:** Plasma triacylglycerols concentration (μM) in six Quaker parrots (*Myiopsitta monachus*) determined by mass spectrometry.

Lipids	Median	IQR	Min	Max	%
TG(44:1)	2.095	0.42	0.907	2.76	0.1
TG(44:2)	1.255	1.318	0.027	1.83	0.0
TG(44:4)	0.012	0.014	0.007	2.16	0.0
TG(46:2)	2.31	0.737	1.42	3.05	0.1
TG(48:1)	22.65	8.35	14.5	34.8	0.6
TG(48:2)	22.25	7.225	17.3	27.1	0.6
TG(48:3)	7.125	2.3	4.34	9.25	0.2
TG(49:1)	2.19	0.84	1.27	2.9	0.1
TG(49:2)	1.96	0.615	1.38	3.03	0.1
TG(50:1)	99.2	40.95	51.2	137	2.6
TG(50:2)	130.5	56.125	75.7	170	3.5
TG(50:3)	56.4	17.8	33.9	75.6	1.5
TG(50:4)	15.5	2.925	10.4	18.5	0.4
TG(51:1)	0.018	1.053	0.015	2.44	0.0
TG(51:2)	4.785	1.757	3	6.27	0.1
TG(51:3)	4.79	1.2	2.59	6.29	0.1
TG(51:4)	3.485	0.893	2.02	4.49	0.1
TG(51:5)	1.685	NA	<LOD	2.45	0.0
TG(52:2)	488.5	190.25	321	726	12.9
TG(52:3)	678	143	438	897	18.0
TG(52:4)	330	54.25	182	413	8.7
TG(52:5)	79.3	11.725	41	97.3	2.1
TG(52:6)	14.25	2.5	8.33	16.2	0.4
TG(52:7)	4.055	0.265	2.98	4.63	0.1
TG(53:3)	7.14	1.407	4.24	8.69	0.2
TG(53:4)	5.05	1.025	2.96	6.66	0.1
TG(53:5)	3.205	0.818	1.9	3.89	0.1
TG(54:2)	42	34.9	6.19	74.4	1.1
TG(54:3)	259	93.25	123	352	6.9
TG(54:4)	504.5	158	212	730	13.4
TG(54:5)	490	130.75	206	727	13.0
TG(54:6)	269.5	55	123	388	7.1
TG(54:7)	70.3	15.3	32.1	103	1.9
TG(55:6)	2.28	0.507	1.66	2.64	0.1
TG(55:7)	1.51	1.29	0.034	2.23	0.0
TG(56:6)	31.85	7.225	23.4	38	0.8
TG(56:7)	60.95	22.6	43.3	74.8	1.6
TG(56:8)	56.1	24.825	37.3	70.8	1.5

IQR, interquartile range; NA, not applicable; LOD, limits of detection.

Diacylglycerols (Table 10) and some ether-linked diarylglycerols (Table 11) were quantified and were present at substantially lower concentrations than TGs. They are metabolic intermediates and metabolites of TGs and glycerophospholipids. DGs in C36 were the most common, likely containing either a combination of C18 fatty acids (stearic, oleic, linoleic, linolenic) or a combination of palmitic acid and arachidonic acid. DGs were identified at the sum composition level of information and it is unknown whether they were 1,2 DG or 1,3 DG. They were not detected through untargeted lipidomics.

**Table 10:** Plasma diacylglycerols concentration (μM) in six Quaker parrots (*Myiopsitta monachus*) determined by mass spectrometry.

Lipids	Median	IQR	Min	Max	%
DG(32:1)	1.375	0.403	0.85	2.2	1.4
DG(32:2)	1.365	0.638	0.934	2.03	1.4
DG(34:1)	9.865	3.043	7.69	13.4	10.1
DG(34:3)	2.315	0.757	1.45	3.09	2.4
DG(36:2)	20.4	5.325	15.9	28.4	20.8
DG(36:3)	31.95	9.75	21.7	40.1	32.6
DG(36:4)	14.65	4.45	9.1	20.8	14.9
DG(38:0)	0.061	NA	<LOD	4	0.1
DG(38:5)	6.675	1.51	4.61	7.23	6.8
DG(39:0)	1.745	0.565	0.929	2.08	1.8
DG(41:1)	6.12	1.365	3.45	6.73	6.2
DG(42:1)	0.976	0.253	0.677	1.12	1.0
DG(42:2)	0.613	0.347	0.582	1.18	0.6
DG(44:3)	0.003	NA	<LOD	2.29	0.0

IQR, interquartile range; NA, not applicable; LOD, limits of detection.

**Table 11:** Plasma acylalkylglycerols concentration (μM) in six Quaker parrots (*Myiopsitta monachus*) determined by mass spectrometry.

Lipids	Median	IQR	Min	Max	%
DG-O(32:2)	3.005	0.993	2.01	3.72	9.5
DG-O(34:1)	23	6.65	18	29.6	72.7
DG-O(36:4)	5.615	0.72	4.44	7.67	17.8

IQR, interquartile range; NA, not applicable; LOD, limits of detection.

Monoacylglycerols were not quantified by targeted techniques, but untargeted methods tentatively identified MG(18:1) and MG(18:2) as common plasmatic species.

#### Glycerophospholipids

Only glycerophosphocholines were quantified as part of targeted lipidomics panels, but untargeted methods detected a large number of other glycerophospholipids (Table 4). Glycerophosphocholines (PCs) was one of the most diverse lipid categories and also included the highest number of lipid species quantified by targeted panels (Tables 12–14). Phosphatidylcholines were the most common PCs especially the species in C34, C36 and C38. As they only contain 2 fatty acid chains, the most likely components were palmitic acid, stearic acid, linoleic acid, linolenic acid, and arachidonic acid. Based on untargeted lipidomics, PE are also common and abundant species in parrot plasma with PE(36:2) common [as for PC(36:2)], but those could not be confirmed and further quantified by targeted techniques.

**Table 12:** Plasma diacylglycerophosphocholines (phosphatidylcholines) concentration (μM) in six Quaker parrots (*Myiopsitta monachus*) determined by mass spectrometry.

Lipids	Median	IQR	Min	Max	%
PC(30:0)	1.6	0.235	1.29	1.97	0.0
PC(31:0)	0.282	0.109	0.223	0.424	0.0
PC(31:1)	0.076	0.037	0.034	0.117	0.0
PC(32:0)	53.55	10.425	39	67.2	1.3
PC(32:1)	32.65	17.075	12.6	39.1	0.8
PC(32:2)	5.51	0.885	4.35	5.95	0.1
PC(32:6)	0.943	0.232	0.564	0.994	0.0
PC(33:0)	1.019	0.193	0.782	1.33	0.0
PC(33:1)	1.95	0.762	1.31	2.36	0.0
PC(33:2)	1.655	0.245	1.54	1.99	0.0
PC(33:4)	0.216	NA	<LOD	1.26	0.0
PC(34:1)	865.5	187.25	519	976	20.2
PC(34:2)	893	138.5	684	945	20.9
PC(34:3)	23.9	7.3	16.2	30.8	0.6
PC(34:4)	2.1	0.865	1.33	2.63	0.0
PC(35:0)	0.209	NA	<LOD	0.962	0.0
PC(35:1)	4.145	1.28	2.91	4.94	0.1
PC(35:2)	8.17	1	6.18	9.23	0.2
PC(35:3)	0.838	0.187	0.512	0.944	0.0
PC(35:4)	0.46	0.109	0.345	0.78	0.0
PC(36:1)	6.255	3.42	1.4	8.37	0.1
PC(36:2)	978	211	749	1053	22.8
PC(36:3)	252.5	59	161	283	5.9
PC(36:4)	285.5	93.75	149	327	6.7
PC(36:5)	30.1	8.8	23.2	49.3	0.7
PC(36:6)	1.865	0.37	1.38	2.03	0.0
PC(37:1)	1.61	0.555	1.11	2	0.0
PC(37:2)	6.815	1.96	5.34	8.96	0.2
PC(37:3)	0.826	0.348	0.535	1.14	0.0
PC(37:4)	5.04	1.322	2.8	5.95	0.1
PC(37:5)	8.43	2.688	5.69	9.67	0.2
PC(37:6)	1.995	0.24	1.46	3.44	0.0
PC(38:1)	2.51	0.663	1.59	3.35	0.1
PC(38:2)	10.65	2.643	6.9	12	0.2
PC(38:4)	437.5	148.75	331	572	10.2
PC(38:5)	74.6	12.65	30.4	93.5	1.7
PC(38:6)	74	7.025	55.5	80.6	1.7
PC(38:7)	29.7	7.85	20.9	38.9	0.7
PC(39:2)	0.572	0.106	0.422	0.658	0.0
PC(39:3)	0.55	0.167	0.043	0.922	0.0
PC(39:4)	1.625	0.52	1.37	2.07	0.0
PC(39:5)	1.165	0.35	0.868	1.92	0.0
PC(39:6)	3.97	1.428	2.33	5.34	0.1
PC(39:7)	1.74	0.725	0.572	2.11	0.0
PC(40:1)	0.973	0.254	0.669	1.12	0.0
PC(40:2)	2.77	0.742	1.97	2.98	0.1
PC(40:3)	0.281	NA	<LOD	1.2	0.0
PC(40:4)	15.4	2.275	11.2	18.3	0.4
PC(40:5)	16.7	3.6	12.6	19.6	0.4
PC(40:6)	91.85	17.525	62.6	110	2.1
PC(40:7)	7.645	11.863	<LOD	21.5	0.2
PC(40:8)	9.04	NA	<LOD	31.2	0.2
PC(40:9)	8.96	3.745	6.1	12.3	0.2
PC(41:2)	0.158	NA	<LOD	0.974	0.0
PC(41:3)	0.034	NA	<LOD	0.447	0.0
PC(41:4)	0.117	0.144	0.032	0.63	0.0
PC(41:5)	0.514	0.119	0.362	0.624	0.0
PC(41:8)	0.253	NA	<LOD	0.436	0.0
PC(42:10)	1.19	NA	<LOD	4.42	0.0
PC(42:2)	0.238	NA	<LOD	0.829	0.0
PC(42:4)	1.147	0.52	0.861	1.44	0.0
PC(42:5)	0.574	0.236	0.417	0.782	0.0
PC(42:6)	1.06	0.222	0.83	1.49	0.0
PC(42:7)	0.85	0.672	0.315	1.67	0.0
PC(43:6)	1.59	0.383	1.05	1.73	0.0
PC(44:10)	0.613	NA	<LOD	0.921	0.0
PC(44:5)	0.968	0.464	0.601	1.51	0.0
PC(44:6)	1.101	0.908	0.137	1.57	0.0
PC(44:7)	1.004	0.584	0.006	2.15	0.0
PC(46:1)	0.022	0.028	0.014	0.143	0.0
PC(46:2)	0.867	0.305	0.617	1.08	0.0

IQR, interquartile range; NA, not applicable; LOD, limits of detection.

**Table 13:** Plasma alkylacylglycerophosphocholines concentration (μM) in six Quaker parrots (*Myiopsitta monachus*) determined by mass spectrometry.

Lipids	Median	IQR	Min	Max	%
PC-O(26:0)	0.083	NA	<LOD	0.157	0.0
PC-O(30:0)	0.197	0.004	0.159	0.231	0.1
PC-O(32:0)	8.745	2.088	8.61	12.2	3.2
PC-O(32:1)	5.605	1.628	5.19	7.72	2.0
PC-O(32:2)	0.309	0.091	0.241	0.419	0.1
PC-O(33:2)	0.595	0.04	0.478	0.841	0.2
PC-O(33:3)	0.43	0.086	0.367	0.497	0.2
PC-O(34:0)	2.215	0.678	1.94	2.96	0.8
PC-O(34:1)	25.15	8.175	23	36.1	9.2
PC-O(34:2)	25.25	9.2	23.3	36	9.2
PC-O(34:3)	9.18	1.892	8.17	10.3	3.3
PC-O(34:4)	0.414	0.42	0.318	1.2	0.2
PC-O(35:3)	0.503	0.763	0.112	1.85	0.2
PC-O(35:4)	1.23	0.676	0.24	1.56	0.4
PC-O(36:1)	2.58	0.608	2.35	3.5	0.9
PC-O(36:2)	18.6	6.6	17.9	27.8	6.8
PC-O(36:3)	16.65	6.15	14.5	23.8	6.1
PC-O(36:4)	25.25	11.1	20.3	36.1	9.2
PC-O(36:5)	19.95	7.525	14.2	25.8	7.3
PC-O(38:1)	0.462	0.366	0.003	1.09	0.2
PC-O(38:2)	1.11	0.449	0.885	1.6	0.4
PC-O(38:3)	<LOD	NA	<LOD	1.33	0.0
PC-O(38:4)	26.25	9.6	22	40	9.6
PC-O(38:5)	41.45	14.725	38.5	58.6	15.1
PC-O(38:6)	12.5	2.775	10.8	17.5	4.5
PC-O(40:2)	0.953	0.544	0.51	1.44	0.3
PC-O(40:3)	0.735	NA	<LOD	0.86	0.3
PC-O(40:4)	3.85	1.59	3.32	6.15	1.4
PC-O(40:5)	7.655	2.865	6.57	10.6	2.8
PC-O(40:6)	6.785	2.345	5.38	10.2	2.5
PC-O(40:7)	4.9	0.835	3.85	5.61	1.8
PC-O(40:8)	0.766	0.603	0.432	1.78	0.3
PC-O(42:4)	0.535	0.165	0.292	0.84	0.2
PC-O(42:5)	2.62	1.112	1.72	3.63	1.0
PC-O(42:6)	1.3	0.683	0.614	1.73	0.5

IQR, interquartile range; NA, not applicable; LOD, limits of detection.

**Table 14:** Plasma monoacylglycerophosphocholines (lysophosphatidylcholines) and monoalkylglycerophosphocholines concentration (μM) in six Quaker parrots (*Myiopsitta monachus*) determined by mass spectrometry.

Lipids	Median	IQR	Min	Max	%
LPC(15:0)	0.158	0.017	0.141	0.169	0.0
LPC(16:0)	121	16.75	98.7	134	19.5
LPC(16:1)	2.44	1.285	1.23	3.24	0.4
LPC(17:0)	1.004	0.072	0.825	1.08	0.2
LPC(17:1)	0.15	0.014	0.14	0.172	0.0
LPC(18:0)	239.5	48	177	271	38.6
LPC(18:1)	132	42.675	86.2	151	21.3
LPC(18:2)	117	17.75	86.7	136	18.8
LPC(20:1)	1.145	0.319	0.721	1.44	0.2
LPC(20:2)	0.581	0.095	0.457	0.621	0.1
LPC(22:5)	0.686	0.106	0.608	0.849	0.1
LPC(22:6)	4.605	1.018	3.03	5.23	0.7
LPC(24:0)	0.48	0.214	0.342	0.62	0.1
LPC-O(16:1)	0.856	0.029	0.75	0.959	0.1
LPC-O(18:0)	1.59	0.39	1.42	2.11	0.3
LPC-O(18:1)	2.84	0.568	2.45	3.56	0.5
LPC-O(18:2)	1.25	0.158	1.23	1.61	0.2

IQR, interquartile range.

Ether-linked PCs (PC-O) were also common and quantified (Table 13).

Lysophophatidylcholines (LPCs) are reported in Table 4 along with LPC-O and are metabolites of PCs. LPCs in C18 and with palmitic acid were the most abundant.

#### Sphingolipids

Most sphingolipids had sphingosine (d18:1) as the sphingoid base; however only sphingolipids with sphingosine or sphinganine (d18:0) were quantified by targeted methods and only the sum composition was obtained for untargeted methods. Sphingosine was also the most abundant non-esterified sphingoid base in the plasma at 87.2% (Table 15).

**Table 15:** Plasma non-esterified sphingoid bases concentration (μM) in six Quaker parrots (*Myiopsitta monachus*) determined by mass spectrometry.

Lipids	Median	IQR	Min	Max	%
Sphinganine (d14:0)	<LOD	NA	<LOD	0.001	0.0
Sphinganine (d16:0)	0.002	0.002	0.001	0.003	0.3
Sphinganine (d17:0)	0.017	0.004	0.013	0.021	2.7
Sphinganine (d18:0)	0.059	0.011	0.037	0.103	9.3
Sphinganine (d18:1)	0.551	0.146	0.351	0.939	87.2
Sphinganine (d18:2)	<LOD	NA	<LOD	<LOD	0.0
Sphinganine (d20:0)	0.002	0.001	0.002	0.004	0.3
Sphinganine (d20:4)	0.001	0	0.001	0.001	0.2

IQR, interquartile range; NA, not applicable; LOD, limits of detection.

Sphingomyelins (SM) were the most abundant plasma sphingolipids and sphingomyelin with long-chain saturated fatty acids were the preponderant species such as the most common, palmitoyl sphingomyelin [SM(d18:1/16:0)]. Saturated fatty acid sphingomyelins accounted for 81.8% of all SMs (Table 16).

**Table 16:** Plasma ceramide phosphocholines (sphingomyelins) concentration (μM) in six Quaker parrots (*Myiopsitta monachus*) determined by mass spectrometry.

Lipids	Median	IQR	Min	Max	%
SM(d18:0/12:0)	<LOD	NA	<LOD	<LOD	0.0
SM(d18:0/16:0)	3.031	0.288	2.512	3.465	1.5
SM(d18:0/24:0)	20.648	1.94	15.863	28.62	10.3
SM(d18:1/06:0)	0.029	0.012	0.024	0.043	0.0
SM(d18:1/12:0)	0.323	0.061	0.288	0.427	0.2
SM(d18:1/14:0)	1.102	0.191	0.969	1.487	0.5
SM(d18:1/16:0)	72.476	8.561	60.734	81.667	36.0
SM(d18:1/17:0)	0.695	0.113	0.632	0.837	0.3
SM(d18:1/18:0)	28.217	3.275	20.861	30.426	14.0
SM(d18:1/18:1)	0.1	0.01	0.076	0.122	0.0
SM(d18:1/18:2)	0.168	0.018	0.156	0.184	0.1
SM(d18:1/20:0)	17.702	1.696	16.409	21.183	8.8
SM(d18:1/20:1)	35.209	4.768	31.681	44.868	17.5
SM(d18:1/20:4)	0.073	0.011	0.045	0.08	0.0
SM(d18:1/22:0)	20.5	2.067	15.518	22.438	10.2
SM(d18:1/22:6)	1.126	0.085	1.044	1.247	0.6

IQR, interquartile range; NA, not applicable; LOD, limits of detection.

Ceramides and dihydroceramides were in much lower concentrations in the plasma than SMs at about 3% of all measured sphingolipids. There was a tendency for these lipids to have very long chain fatty acids (>C22) as esterified fatty acids. The most common ceramide included nervonic acid [Cer(d18:1/24:1)] (Table 17). Dihydroceramides were in minute concentrations when compared to ceramides (Table 18).

**Table 17:** Plasma ceramides concentration (μM) in six Quaker parrots (*Myiopsitta monachus*) determined by mass spectrometry.

Lipids	Median	IQR	Min	Max	%
Cer(d18:1/2:0)	0.03	0.008	0.019	0.035	0.5
Cer(d18:1/4:0)	0.022	0.016	0.015	0.046	0.4
Cer(d18:1/6:0)	0.005	0.001	0	0.015	0.1
Cer(d18:1/8:0)	<LOD	NA	<LOD	<LOD	0.0
Cer(d18:1/10:0)	0.003	0.002	0.002	0.007	0.1
Cer(d18:1/12:0)	0.001	NA	<LOD	0.003	0.0
Cer(d18:1/14:0)	0.002	0	0.002	0.002	0.0
Cer(d18:1/16:0)	0.064	0.151	0.007	0.245	1.1
Cer(d18:1/17:0)	<LOD	NA	<LOD	<LOD	0.0
Cer(d18:1/18:0)	0.001	0.001	0.001	0.002	0.0
Cer(d18:1/18:1)	<LOD	NA	<LOD	<LOD	0.0
Cer(d18:1/20:0)	0.01	0.003	0.006	0.012	0.2
Cer(d18:1/20:4)	<LOD	NA	<LOD	<LOD	0.0
Cer(d18:1/22:0)	0.783	0.16	0.675	1.387	13.1
Cer(d18:1/23:0)	1.16	0.401	0.767	1.56	19.3
Cer(d18:1/24:0)	1.258	0.207	0.942	1.974	21.0
Cer(d18:1/24:1)	2.609	2.237	1.078	5.3	43.5
Cer(d18:1/25:0)	0.036	NA	<LOD	0.368	0.6
Cer(d18:1/26:0)	0.012	0.003	0.007	0.018	0.2
Cer(d18:1/28:0)	0.001	0	0.001	0.001	0.0

IQR, interquartile range; NA, not applicable; LOD, limits of detection.

**Table 18:** Plasma dihydroceramides concentration (μM) in six Quaker parrots (*Myiopsitta monachus*) determined by mass spectrometry.

Lipids	Median	IQR	Min	Max	%
Dihydroceramide(d18:0/12:0)	<LOD	NA	<LOD	<LOD	0.0
Dihydroceramide(d18:0/14:0)	<LOD	NA	<LOD	<LOD	0.0
Dihydroceramide(d18:0/16:0)	0.001	0	0.001	0.001	2.4
Dihydroceramide(d18:0/17:0)	<LOD	NA	<LOD	<LOD	0.0
Dihydroceramide(d18:0/18:0)	<LOD	NA	<LOD	<LOD	0.0
Dihydroceramide(d18:0/18:1)	<LOD	NA	<LOD	<LOD	0.0
Dihydroceramide(d18:0/18:2)	<LOD	NA	<LOD	<LOD	0.0
Dihydroceramide(d18:0/2:0)	<LOD	NA	<LOD	0.001	0.0
Dihydroceramide(d18:0/20:0)	0.002	0.001	0.002	0.003	4.9
Dihydroceramide(d18:0/20:4)	<LOD	NA	<LOD	<LOD	0.0
Dihydroceramide(d18:0/22:0)	0.005	0.001	0.004	0.006	12.2
Dihydroceramide(d18:0/22:6)	<LOD	NA	<LOD	0.001	0.0
Dihydroceramide(d18:0/24:0)	0.033	0.014	0.02	0.042	80.5

IQR, interquartile range; NA, not applicable; LOD, limits of detection.

A few cerebrosides (monoglycosylceramides) and globosides (polyglycosylceramides) were quantified (Table 19). Like ceramides and dihydroceramides, they had very long chain fatty acids.

**Table 19:** Plasma simple glycosphingolipids (cerebrosides and globosides) concentration (μM) in six Quaker parrots (*Myiopsitta monachus*) determined by mass spectrometry.

Lipids	Median	IQR	Min	Max	%
Galactosyl(beta)ceramide(d18:1/12:0)	0.002	0.001	0.001	0.004	0.2
Galactosyl(beta)ceramide(d18:1/16:0)	0.024	0.005	0.018	0.031	1.9
Galactosyl(beta)ceramide(d18:1/22:0)	0.264	0.052	0.167	0.276	20.4
Galactosyl(beta)ceramide(d18:1/24:0)	0.613	0.252	0.434	0.893	47.3
Galactosyl(beta)ceramide(d18:1/24:1)	0.323	0.105	0.25	0.475	24.9
Glucosyl(beta)ceramide (d18:1/18:0)	0.005	0.001	0.005	0.009	0.4
Glucosyl(beta)ceramide(d18:1/18:1)	0.001	NA	<LOD	0.002	0.1
Lactosyl(beta)ceramide(d18:1/16:0)	0.065	0.011	0.055	0.075	5.0
Lactosyl(beta)ceramide(d18:1/18:1)	<LOD	NA	<LOD	<LOD	0.0

IQR, interquartile range; NA, not applicable; LOD, limits of detection.

#### Sterol lipids

As mentioned above, sterol lipids, in particular free cholesterol and CE(18:2) dominate the plasma lipidome of the Quaker parrot. Cholesteryl esters (CE) (Table 20) compose about 2/3 of plasma cholesterol with the remaining 1/3 being free cholesterol (Table 21). Most important CE had polyunsaturated fatty acids such as linoleic acid, linolenic acid, arachidonic acid, and DHA.

**Table 20:** Plasma cholesteryl esters concentration (μM) in six Quaker parrots (*Myiopsitta monachus*) determined by mass spectrometry.

Lipids	Median	IQR	Min	Max	%
CE(16:0)	479.5	113	361	521	3.3
CE(16:1)	198.5	121.55	91.1	265	1.4
CE(17:0)	11	1.595	7.07	13.1	0.1
CE(17:1)	12.7	3.715	7.07	13.8	0.1
CE(17:2)	6.355	0.715	4.27	8.09	0.0
CE(18:1)	465	94	243	565	3.2
CE(18:2)	10419.5	2672.75	7704	12122	72.5
CE(18:3)	668	173.75	482	752	4.6
CE(19:2)	175.5	40.5	101	226	1.2
CE(19:3)	57.3	15.425	30.3	68.6	0.4
CE(20:4)	960	302.75	707	1377	6.7
CE(20:5)	202	64.75	120	264	1.4
CE(22:5)	151.5	80.75	116	261	1.1
CE(22:6)	562	162	401	674	3.9

IQR, interquartile range.

**Table 21:** Plasma sterols and steroids concentration (μM) in six Quaker parrots (*Myiopsitta monachus*) determined by mass spectrometry.

Lipids	Median	IQR	Min	Max
6bOH-cortisol	<LOD	NA	<LOD	<LOD
7-Dehydrocholesterol	0.042	0.007	0.034	0.068
Aldosterone	0.001	NA	<LOD	0.002
Androstenedione	0.097	0.016	0.081	0.167
Cholesterol	9438.4	1328.3	7715.8	12134.2
Cortisol	0.002	0.002	0.001	0.003
Dehydrodesmosterol	0.32	0.17	0.238	0.673
Dehydrolathosterol	3.038	0.847	2.229	3.449
Desmosterol	0.509	0.298	0.34	1.194
Dihydrolanosterol	0.054	0.01	0.046	0.078
Lanestenol	<LOD	NA	<LOD	<LOD
Lathosterol	0.268	0.058	0.2	0.512
Zymostenol	<LOD	NA	<LOD	<LOD
Zymosterol	0.109	0.039	0.077	0.24

IQR, interquartile range; NA, not applicable; LOD, limits of detection.

While free cholesterol was the main non-esterified sterol, other species were quantified as well as some steroids, which are synthesized from cholesterol (Table 21). Corticosterone, the most important steroid of birds, was not part of this targeted steroid panel.

A comprehensive bile acid panel was also performed on the plasma of these parrots (Table 22). By far, the most common plasma bile acid of birds was taurochenodeoxycholic acid. Taurocholic acid was the second most common plasma bile acid and together with taurochenodeoxycholic acid represented 87.4% of all bile acids.

**Table 22:** Plasma bile acids concentration (μM) in six Quaker parrots (*Myiopsitta monachus*) determined by mass spectrometry.

Bile acid	Median	IQR	Min	Max	%
12-Ketochenodeoxycholic acid	<LOD	NA	<LOD	0.001	0.0
12-Ketolithocholic acid	<LOD	NA	<LOD	0.001	0.0
3-Oxocholic acid	<LOD	NA	<LOD	<LOD	0.0
3b-OH-5-cholestenoic acid	0.106	0.064	0.073	0.245	0.3
3b7a-diOH-5-cholestenoic acid	0.028	0.006	0.024	0.036	0.1
6,7-Diketolithocholic acid	0.001	NA	<LOD	0.001	0.0
7-Ketodeoxycholic acid	<LOD	NA	<LOD	<LOD	0.0
7-Ketolithocholic acid	<LOD	NA	<LOD	<LOD	0.0
7aOH-3-oxo-4-cholestenoic acid	0.128	0.009	0.106	0.134	0.4
α-Muricholic acid	<LOD	NA	<LOD	<LOD	0.0
Allocholic acid	0.001	NA	<LOD	0.002	0.0
Allocholic acid-3-Sulfate	<LOD	NA	<LOD	<LOD	0.0
Alloisolithocholic acid	0.001	NA	<LOD	0.001	0.0
Apocholic acid	<LOD	NA	<LOD	<LOD	0.0
β-Muricholic acid	<LOD	NA	<LOD	<LOD	0.0
Chenodeoxycholic acid	0.008	0.001	0.007	0.009	0.0
chenodeoxycholic acid-24-glucuronide	<LOD	NA	<LOD	<LOD	0.0
chenodeoxycholic acid-3-glucuronide	<LOD	NA	<LOD	<LOD	0.0
chenodeoxycholic acid-3-Sulfate	<LOD	NA	<LOD	<LOD	0.0
Cholic acid	<LOD	NA	<LOD	0.001	0.0
cholic acid-3-Sulfate	<LOD	NA	<LOD	<LOD	0.0
Dehydrocholic acid	<LOD	<LOD	<LOD	<LOD	0.0
Dehydrolithocholic acid	<LOD	NA	<LOD	0.001	0.0
Deoxycholic acid	0.001	NA	<LOD	0.008	0.0
deoxycholic acid-24-glucuronide	<LOD	NA	<LOD	<LOD	0.0
deoxycholic acid-3-glucuronide	<LOD	NA	<LOD	<LOD	0.0
deoxycholic acid-3-Sulfate	<LOD	NA	<LOD	<LOD	0.0
Dioxolithocholic acid	<LOD	NA	<LOD	<LOD	0.0
Glycoallocholic acid-3-sulfate	<LOD	NA	<LOD	<LOD	0.0
Glycochenodeoxycholic acid	0.001	0	0.001	0.002	0.0
Glycochenodeoxycholic acid-3Sulfate	<LOD	NA	<LOD	<LOD	0.0
Glycocholic acid	<LOD	NA	<LOD	0.001	0.0
Glycocholic acid-3-sulfate	<LOD	NA	<LOD	<LOD	0.0
Glycodehydrocholic acid	<LOD	NA	<LOD	<LOD	0.0
Glycodeoxycholic acid	<LOD	NA	<LOD	0.001	0.0
Glycodeoxycholic acid-3Sulfate	<LOD	NA	<LOD	<LOD	0.0
Glycohyocholic acid	<LOD	NA	<LOD	<LOD	0.0
Glycohyodeoxycholic acid	<LOD	NA	<LOD	<LOD	0.0
Glycolithocholic acid	<LOD	NA	<LOD	0.001	0.0
GlycolithoCholic acid-3Sulfate	<LOD	NA	<LOD	<LOD	0.0
Glycoursodeoxycholic acid	<LOD	NA	<LOD	0.001	0.0
Glycoursodeoxycholic acid-3-Sulfate	<LOD	NA	<LOD	<LOD	0.0
Hyodeoxycholic acid	<LOD	NA	<LOD	<LOD	0.0
Isodeoxycholic acid	<LOD	NA	<LOD	<LOD	0.0
Isolithocholic acid	0.001	NA	<LOD	0.001	0.0
Lithocholic acid	0.001	NA	<LOD	0.001	0.0
lithoCholic acid-24-glucuronide	<LOD	NA	<LOD	0.001	0.0
lithoCholic acid-3-glucuronide	<LOD	NA	<LOD	<LOD	0.0
lithoCholic acid-3-Sulfate	<LOD	NA	<LOD	<LOD	0.0
Murocholic acid	<LOD	NA	<LOD	<LOD	0.0
γ-muricholic acid	<LOD	NA	<LOD	<LOD	0.0
Norcholic acid	<LOD	NA	<LOD	<LOD	0.0
Nordeoxycholic acid	<LOD	NA	<LOD	<LOD	0.0
Norursodeoxycholic acid	<LOD	NA	<LOD	<LOD	0.0
Tauro-a-muricholic acid	0.041	0.018	0.028	0.099	0.1
Tauro-b-muricholic acid	<LOD	NA	<LOD	<LOD	0.0
Tauro-w-muricholic acid	1.286	0.356	0.985	3.764	4.2
Tauroallocholic acid	2.046	0.55	1.214	4.629	6.7
Taurochenodeoxycholic acid	21.195	12.768	14	46.36	68.9
Taurochenodeoxycholic acid-3-sulfate	0.01	0.006	0.004	0.016	0.0
Taurocholic acid	5.675	2.828	3.525	11.86	18.5
Taurodehydrocholic acid	<LOD	NA	<LOD	<LOD	0.0
Taurodeoxycholic acid	0.003	0.002	0.002	0.005	0.0
Taurodeoxycholic acid-3-sulfate	<LOD	NA	<LOD	<LOD	0.0
Taurohyocholic acid	0.002	0.001	0.001	0.003	0.0
Taurolithocholic acid	0.141	0.064	0.093	0.186	0.5
TaurolithoCholic acid-3-sulfate	0.073	0.05	0.032	0.188	0.2
Tauroursodeoxycholic acid-3-sulfate	<LOD	NA	<LOD	<LOD	0.0
Tauroursodexycholic/Taurohyodeoxycholic acid	0.02	0.008	0.014	0.034	0.0
Ursocholic acid	<LOD	NA	<LOD	<LOD	0.0
Ursodeoxycholic acid	<LOD	NA	<LOD	0.001	0.0
ursodeoxycholic acid-24-glucuronide	<LOD	NA	<LOD	<LOD	0.0
ursodeoxycholic acid-3-glucuronide	<LOD	NA	<LOD	<LOD	0.0
ursodeoxycholic acid-3-Sulfate	<LOD	NA	<LOD	<LOD	0.0
w-muricholic acid	<LOD	NA	<LOD	<LOD	0.0

IQR, interquartile range; NA, not applicable; LOD, limits of detection.

#### Others

Other metabolites associated with lipid metabolism were quantified as part of the targeted lipidomics panels. These included dicarboxylic acids important in the TCA cycle (which provides substrates for fatty acid and cholesterol biosynthesis), free carnitine and other lipid-like metabolites (Table 23).

**Table 23:** Plasma concentration (μM) of non-lipid or lipid-like metabolites and organic acids important in fatty acid metabolic pathways in six Quaker parrots (*Myiopsitta monachus*) determined by mass spectrometry.

Analytes	Median	IQR	Min	Max
γ-Butyrobetaine	0.274	0.073	0.205	0.423
Free carnitine	3.786	0.398	3.223	4.605
α-Ketoglutaric acid	121.75	32.02	88.23	145.50
Citric acid	281.9	43.85	213.2	341.9
Fumaric acid	23.82	7.9	16.63	31.17
Glycolic acid	6.69	1.84	4.96	9.01
Isocitric acid	8.55	1.82	5.94	12.86
Lactic acid	11190	4860	9119	18730
Malic acid	101.39	29.86	63.60	137.70
Pyruvic acid	620.9	162.27	415.7	786.9
Succinic acid	57.05	23.23	25.86	89.99

IQR, interquartile range.

### Sex differences

**Fig 2:**
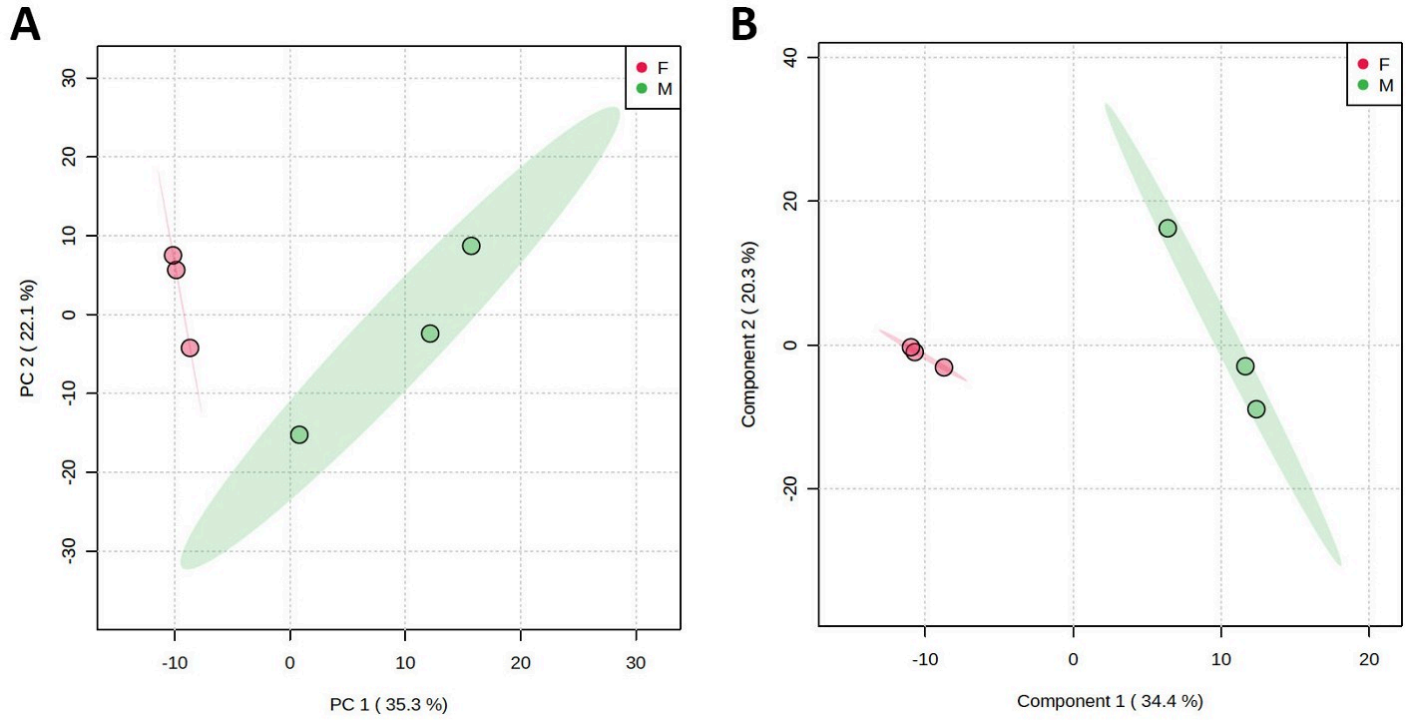
PCA scores plot (A) and PLS-DA scores plot (B) between the first two components showing clustering of male and female Quaker parrots (*Myiopsitta monachus*) using targeted lipidomics panel between sexes. Grouping is shown as different colors with their 95% confidence ellipses. The explained variances are shown in brackets.

On serial t-tests for targeted results on lipid concentrations, only 2 lipid species, both minor PCs (see Tables 12 and 13), were found to be significantly different between sexes, PC(42:4) (q = 0.024) and PC-O(34:3) (q = 0.024). The sexes were well clustered on PCA and PLS-DA plots without overlap (Fig 2). The PCA biplot was difficult to interpret because of the number of variables. Using VIP scores, the most important discriminating variables between sexes on PLS-DA were mainly PCs [PC(42:4) and PC-O(34:3) being the most important], acyl-carnitines (C12:1, C14:0, and C12:0 being the most important), and TG(56:7) for females, and Cer(d18:1/18:0) for males. The PCA model explained 57.4% of the variance and the PLS-DA model explained 54.7% of the variance.

**Fig 3:**
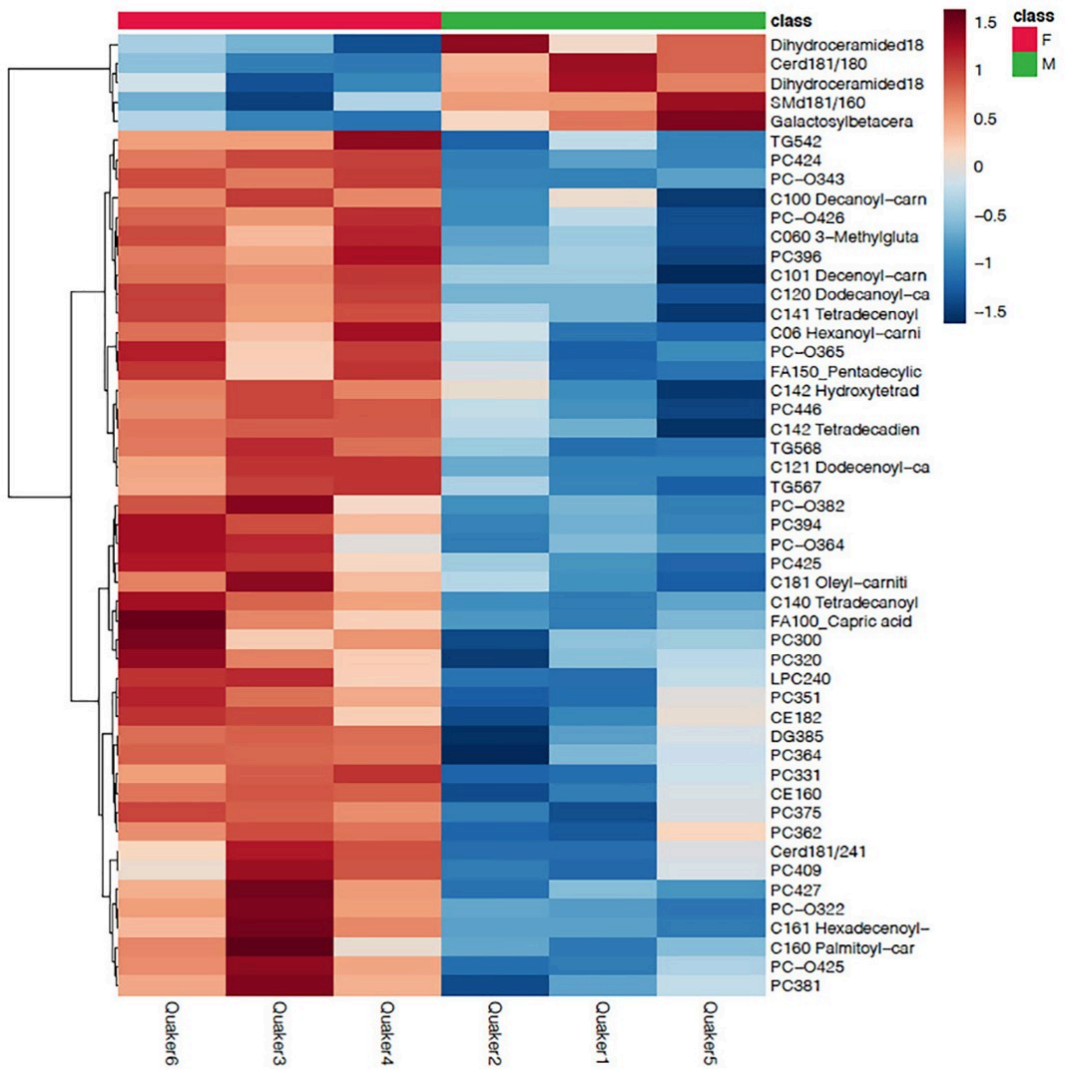
Heatmap showing clustering of lipid species from targeted lipidomics panels between sexes in Quaker parrots (*Myiopsitta monachus*). A clustering dendrogram is also present on the left, the different parrots are on the x-axis and the lipid analytes on the y-axis. Only the 50 most important lipids based on their t-test p-values are displayed. It should be noted that most of these lipids did not show significant differences between sexes on univariate analysis. Color coding represents fold changes on normalized plasma concentrations.

The heatmap also suggested different lipidome profiles between sexes (Fig 3). Most lipids were in higher plasma concentrations in female parrots, in particular glycerophospholipids, acyl-carnitines, and some TGs and CEs. A few lipid species were higher in males, all being sphingolipids.

## Discussion

This report presents the first comprehensive database of plasma lipid species in a psittacine bird. The plasma lipidome of animals is astoundingly diverse and several quantitative or semi-quantitative mass spectrometric methods are typically needed to grasp a portion of this diversity due to the various molecular structures and abundance of the different lipid categories [21, 41, 42]. While the targeted lipidomics panels used here give a good overview of the plasma lipidome of the Quaker parrot, only a fraction of the plasma lipids were quantified. As some lipids are structurally complex, we only reported individual lipid species or group of species at the brutto level (sum composition of carbons and carbon-carbon double bonds) for most complex lipids such as glycerolipids and glycerophospholipids or medio level (with added knowledge of fatty acyl chains) for sphingolipids [29]. For complex lipids with many possible combination of fatty acids such as TG, a single reported species such as displayed in Table 9 may encompass several dozen TG isomeric species (same elemental composition, but different lipids), so this is a limitation of the present study and MS/MS characterization of TG and glycerophospholipids should be considered in the future. However, most likely TG candidates can reasonably be predicted based on known stereochemical structures of these lipids as well as the most common fatty acyl chains present in animals and given that the fatty acid composition of the diet was determined. The impact of diet on the fatty acid composition of plasma TG of several parrot species has been determined [43]. For monoacyl lipids such as non-esterified fatty acids, cholesteryl esters, lyso-PC, the sum composition obviously gives the type of fatty acid. But even then, the position of the double bonds along the fatty acyl chain, the stereochemical positions of the different fatty acyl chains (esterified positions on the glycerol molecule or other head groups), and the configuration of the carbon-carbon double bonds (cis/trans isomers) are unknown and several lipid species are still represented. In some cases, such as for omega-3 or omega-6 fatty acids, the position of these double bonds is clinically relevant. Untargeted lipidomics is semi-quantitative (peak intensity) and is typically used more for exploratory analysis and discovery work as the analysis is unbiased and not restricted to predetermined panels [44]. Identified lipid species must ideally be confirmed and quantified by targeted methods [44]. For each match with lipid databases, many isobaric species (same or nearly same mass, but different lipids) are also possible, so lipid identification is less certain. Nevertheless, it was used here to complement the targeted panels and give a glimpse of the diversity of other lipids present in Quaker parrot plasma and to highlight which lipid groups and species are important.

In addition to the incomplete information on individual lipid species, our investigation of the Quaker parrot lipidome was limited to available targeted panels. In particular, the prenol lipids were not investigated, a group including dolichols, ubiquinones, some fat-soluble vitamins (K and E), and carotenoids. Within investigated lipid categories, some important lipids were also not measured such as most glycerophospholipids other than PC, MGs, and cardiolipins. Several bioactive lipids were reported here, mainly steroid hormones, but the mediator lipidome including fatty acid derivatives was not reported as it was considered outside of the scope of this report, which focused on the high abundance lipid (macrolipidomics). Nevertheless, a complete characterization of the parrot lipidome at a high level of molecular information would require considerable resources and extensive analysis and we believe the snapshot of the lipidome reported here gives a reasonable overview of what could be of relevance for potential clinical research applications. The reported lipidome of other species, mainly mammals, is typically of comparable breadth and details [21, 45, 46]

As the plasma lipidome of other avian species including chickens has not been reported, comparisons can only be made with mammals, in particular humans in which it has been well characterized and quantitative data are available [21, 45–47]. However, methodologies in these other studies were different, thus comparisons were mainly made based on large magnitude of concentration differences and on relative abundance within lipid classes. In addition, the plasma lipidome is heavily influenced by the diet, in particular its fatty acid profile, and the reported lipidome should be interpreted in the context of the diet consumed as humans are omnivorous and Quaker parrots are frugivorous/granivorous. For this reason, the parrot diet was also analyzed. Surprisingly, the Quaker parrot lipidome had many similarities to reported mammalian lipidomes, but also some unique differences. The relative importance of the different lipid categories was similar in parrots and humans on a molar basis (sterols>glycerolipids>glycerophospholipids); however, most lipid group concentrations were much higher in the parrots than in humans [21]. Several very abundant lipids in the human plasma lipidome were also found to be abundant in parrots.

The parrot plasma lipidome was dominated by free cholesterol and cholesteryl linoleate [CE(18:2)] on a molar basis, which is also the case in humans. However, CE(18:2) represents only 50% of all CE in humans compared to more than 70% in parrots, but the same number of CE species weren’t analyzed. Free cholesterol and all CE concentrations were also much higher than in humans. However, lathosterol, a marker of cholesterol synthesis, was much lower in Quaker parrots.

For glycerophospholipids, PC(36:2), PC(34:2), and PC(38:4) were some of the most common PCs in both species. PC species identified as abundant on untargeted techniques were also common species in human plasma. Common LPCs in Quaker parrots were also common in humans.

For glycerolipids, a similar TG profile was also observed with TG(52:3) being the most common plasma TG in both species followed by TGs in C52 or C54. The DG profiles were also similar with DG in C36 being the most common.

For sphingolipids, sphingosine was the most common sphingoid base in Quaker parrot as it is the case in humans and mammals in general. SM(d18:1/16:0) was the most common sphingomyelin by far representing about 1/3 of all SM in both species. It is also the most common SM in a number of other mammals [48]. As in humans, the fatty acid distribution in ceramides was quite different from SM with palmitic acid contributing very little compared to very-long chain fatty acids. As in humans and other mammals plasma, ceramides containing C24:1 and C24:0 were the most abundant ceramides in Quaker parrot plasma representing more than 60% of all ceramides [48]. Ceramides are clinically important metabolites and lipid precursors and can also act as bioactive lipids. They have frequently been identified as potential biomarkers for a variety of diseases in humans and mammalian models [20, 49, 50]. Therefore, these similarities in parrots may translate into similar ceramide profiles for lipid-related diseases. Likewise, glycosphingolipids had similar fatty acid compositions than in humans with very-long chain fatty acids predominating.

A difference observed between humans and Quaker parrots pertain to their non-esterified fatty acid profile. In Quaker parrots, palmitic acid was the most common free fatty acid in plasma by far despite these parrots having a dietary fatty acid profile dominated by oleic acid. Free palmitic acid is a potent mediator of lipotoxicity and high levels in Quaker parrots are noteworthy considering their high susceptibility to hepatic lipidosis [2, 51]. By comparison, oleic acid is the main free fatty acid in human plasma. In addition, all concentrations of free fatty acids were far higher to reported concentrations in humans. However, palmitic, stearic, and oleic acids still represented the vast majority of free fatty acids in both species and arachidonic acid and linoleic acids were also the most common PUFAs in both species.

Another marked difference was in the plasma bile acid profile. In parrots, the two major bile acids representing close to 90% of all bile acids were the taurine-conjugated bile acids taurochenodeoxycholic acid and taurocholic acid. The same bile acids also predominate in chickens and turkeys [52]. In humans, these 2 bile acids are also common in plasma but glycine-conjugated bile acids are equally or more common [53, 54] whereas they were at very low to undetectable concentrations in Quaker parrot plasma.

Different lipidomic profiles were detected in female and male Quaker parrots in this study. Although our analysis lacked statistical power to find significant differences between sexes among the individual lipids species, most lipids still appear to be more abundant in females, especially glycerophospholipids and some acyl-carnitines, TGs, and CEs whereas a number of sphingolipids were more abundant in males. These differences are likely explained by the specific female lipid metabolism associated with egg laying (vitellogenesis). Vitellogenesis is typically associated with increased plasma TG and Glycerophospholipids [55]. The lipids found in eggs are mainly composed of TGs and glycerophospholipids with a lower proportion of cholesterol and cholesteryl esters as shown by published lipidomic analysis of chicken egg yolk [56]. Males also had more Cer(d18:1/18:0), which was identified on both PLS-DA and heatmaps and also more of other long-chain fatty acid sphingolipid species than females. In humans, a number of plasmatic sphingolipids are increased in association with steatohepatitis and fatty liver disease [20]. It is interesting as male Quaker parrots have more than 3 times the prevalence of hepatic lipidosis than female Quaker parrots so this association could also prove to be important in this species [2]. The Quaker parrots in this study were young but either close to sexual maturity or had just reached sexual maturity at time of sampling as some females laid eggs just a few months after the study (sexual maturity is reported to be at 1–2 years of age in this species).

Only 12 Quaker parrots were used in this study with 6 replicates per panel. While this seems like a small sample size, 5–6 animals are typically used for discovery studies on previously uncharacterized plasma lipidomes due to the number of panels and the associated high cost of lipidomic analysis [45–47, 57]. Some of the reported animal plasma lipidomes were also only determined using untargeted techniques. The parrots in this study were relatively young and from a homogeneous colony, therefore the reported lipidome may not capture the variability that is present in different demographic groups and in older parrots.

Another limitation of our study is the accuracy of reported lipid concentrations. While targeted lipidomics is quantitative, it still suffers from accuracy and precision issues. This is due in part to the lack of suitable internal standards for every lipid species, differing ion suppression, collision energy, and ionization efficiency between lipid and fatty acid types, and the lack of standardization of lipidomics techniques [41, 44, 58–60]. Therefore, reported concentrations should be considered as semiquantitative estimates of the true concentrations and comparisons between lipids should only be made within the same lipid classes and using the reported relative values (% in tables or fold changes). Studies have shown up to 30–80% variability/imprecision with various methodology settings and depending on lipid classes [35, 58, 60]. The accuracy of the Biocrates Absolute IDQ p400 HR kit used in our study has specifically been investigated across laboratories and showed some inaccuracies with biases of about 50% for most analytes [35]. In this kit, TGs and CEs had the largest biases up to 100–200% in overestimation [35]. This was definitely observed in this study as total cholesterol compounds (free cholesterol + CEs) and total glycerides (TGs + DGs) were largely overestimated compared to known plasma values in this colony of Quaker parrots as measured using conventional techniques [12]. TGs and CEs are neutral lipids, which present specific challenges for absolute quantification using MS-based lipidomics [61].

In conclusion, this study lays the necessary groundwork for further research on the pathophysiology, biomarkers discovery, and pharmacologic intervention of lipid-related diseases in parrots such as atherosclerosis and hepatic lipidosis, which are exceedingly common in captivity. Lipid biomarkers of lipid-related diseases in humans are often lipid mediators such as those derived from polyunsaturated fatty acids (arachidonic acid, ALA, DHA, EPA) as part of the microlipidome or glycerophospholipids such as PCs, PEs, and PIs or metabolite intermediates and precursors of structural lipids such as non-esterified fatty acids, ceramides, LPCs, LPC-Os, DGs, and a variety of small lipid molecules [20, 22, 41, 49, 62, 63]. In Quaker parrots, some of the relative abundance and importance of these lipids is very similar to humans such as for most PCs and DGs while it is very different for others such as non-esterified fatty acids and ceramides. Future research exploring these lipid biomarkers is therefore likely to find both conserved biomarkers across species and species-specific biomarkers. In addition, specific lipidomic signatures of the various psittacine lipid disorders could be investigated as well as lipidomic profiling of parrot plasma with various nutritional and pharmacological interventions for lipid disorders. Finally, lipidomic fingerprinting between various species of parrots is another area that may help to elucidate species predisposition to certain lipid disorders.
